# The meV XUV-RIXS facility at UE112-PGM1 of BESSY II

**DOI:** 10.1107/S1600577522003551

**Published:** 2022-04-26

**Authors:** Karl Bauer, Jan-Simon Schmidt, Frank Eggenstein, Régis Decker, Kari Ruotsalainen, Annette Pietzsch, Thomas Blume, Chun-Yu Liu, Christian Weniger, Frank Siewert, Jana Buchheim, Grzegorz Gwalt, Friedmar Senf, Peter Bischoff, Lisa Schwarz, Klaus Effland, Matthias Mast, Thomas Zeschke, Ivo Rudolph, Andreas Meißner, Alexander Föhlisch

**Affiliations:** a Institute for Methods and Instrumentation for Synchrotron Radiation Research (PS-ISRR), Helmholtz-Zentrum Berlin für Materialien und Energie GmbH (HZB), Albert-Einstein Straße 15, 12489 Berlin, Germany; b Department for Optics and Beamlines (WI-AOS), Helmholtz-Zentrum Berlin für Materialien und Energie GmbH (HZB), Albert-Einstein Straße 15, 12489 Berlin, Germany; c Department for Experiment Control and Data Acquisition (IT-ED), Helmholtz-Zentrum Berlin für Materialien und Energie GmbH (HZB), Albert-Einstein Straße 15, 12489 Berlin, Germany; d Department for Precision Gratings (WI-APG), Helmholtz-Zentrum Berlin für Materialien und Energie GmbH (HZB), Albert-Einstein Straße 15, 12489 Berlin, Germany; e Department for Accelerator Operation and Technology (BE-IA-AOT), Helmholtz-Zentrum Berlin für Materialien und Energie GmbH (HZB), Albert-Einstein Straße 15, 12489 Berlin, Germany

**Keywords:** resonant inelastic X-ray scattering, X-ray photoelectron spectroscopy, XUV, soft X-ray spectroscopy, BESSY II

## Abstract

Resonant inelastic X-ray scattering in the XUV-regime has been implemented at BESSY II, pushing for a few-meV bandwidth in inelastic X-ray scattering at transition metal *M*-edges, rare earth *N*-edges and the *K*-edges of light elements up to carbon with full polarization control. The new dedicated low-energy beamline UE112-PGM1 has been designed to provide 1 µm vertical and 20 µm horizontal beam dimensions that serve together with sub-micrometre solid-state sample positioning as the source point for a high-resolution plane grating spectrometer and a high-transmission Rowland spectrometer for rapid overview spectra. The design and commissioning results of the beamline and high-resolution spectrometer are presented. Helium autoionization spectra demonstrate a resolving power of the beamline better than 10 000 at 64 eV with a 300 lines mm^−1^ grating while the measured resolving power of the spectrometer in the relevant energy range is 3000 to 6000.

## Abstract 2

Resonant inelastic X-ray scattering in the XUV-regime with few meV bandwidth has been implemented at the dedicated low-energy beamline UE112-PGM1 at BESSY II.

## 1. Introduction

Resonant inelastic X-ray scattering (RIXS) can be used to measure electronic, vibrational, orbital and magnetic excitations in various systems with chemical, symmetry and site selectivity (Gel’mukhanov *et al.*, 2021 ▸). In combination with first principles approaches and model descriptions, essential insights to electronic structure, extended potential energy surfaces as well as dynamic relaxation processes are available. RIXS, as a resonant Raman technique, obeys stringent symmetry selection rules that allow us to exploit polarization-dependent and momentum conservation laws in gaining additional selectivity. Soft X-ray RIXS with a strong focus on the *K*-edges of nitrogen and oxygen as well as the transition metal *L*-edges has become a powerful experimental technique with an extensive body of work over the last 20 years (Gel’mukhanov *et al.*, 2021 ▸; Ament *et al.*, 2011 ▸; Savchenko *et al.*, 2021 ▸; Schmitt *et al.*, 2014 ▸). In contrast the XUV region ending just above the carbon *K*-edge (280 eV) has been much less in the focus of investigation and only very few high-resolution RIXS stations exist for this energy range (Strocov *et al.*, 2010 ▸; Yamane *et al.*, 2013 ▸; Xue *et al.*, 2014 ▸; Schmitt *et al.*, 2013 ▸; Kokkonen *et al.*, 2021 ▸; Dziarzhytski *et al.*, 2020 ▸). In this region access to the *M*-edges of transition metals as well as to the *N-*edges of elements of the lanthanide series is possible in addition to the shallow core levels of highly important elements such as silicon, beryllium, sulfur, phosphor and carbon, to name a few. While the ground and final states are the same in RIXS measurements at the *M*-edge and *L*-edge, several parameters like the core-hole lifetime, spin–orbit interaction and surface sensitivity are different (Chiuzbăian *et al.*, 2008 ▸; Wray *et al.*, 2013 ▸). Therefore, both measurements complement each other to provide a clearer picture of the system under investigation.

At the electron-storage ring BESSY II we have designed and implemented a dedicated confocal XUV RIXS setup, creating a new optimized beamline on the XUV undulator UE112 at its PGM1 branch. This allows us to utilize the existing XUV source properties (Schiwietz *et al.*, 2015 ▸; Bahrdt *et al.*, 2010 ▸) augmented with a high-transmission Rowland spectrometer for rapid overview spectra prior to zooming in to the spectral region of interest with the high-resolution spectrometer meV-RIXS.

## 2. Optical layout

**Figure 1:**
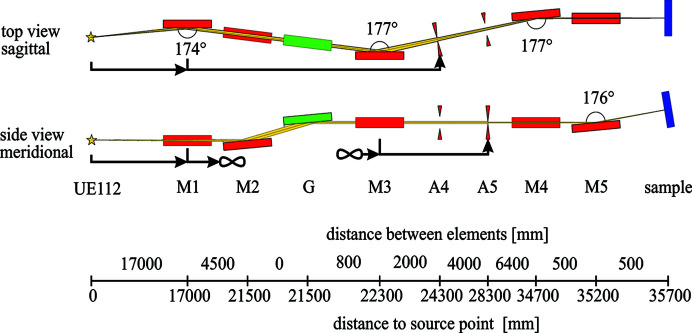
Optical layout of the beamline UE112-PGM1 at BESSY II. Distances are design parameters and are given in millimetres.

The main design goal of the beamline was a 1 µm × 20 µm (vertical × horizontal, FWHM) focus size at the sample to achieve high energy resolution with the meV-RIXS spectrometer (Könnecke *et al.*, 2013 ▸). To achieve this, a horizontal intermediate focus point and better refocusing optics were introduced compared with the old beamline design (Schiwietz *et al.*, 2015 ▸). Furthermore the beamline was moved sideways to accommodate the meV-RIXS spectrometer. As a result we had to replace the mirrors M1, M3, M4 and M5 while the monochromator (M2 and G) stayed the same as shown in the sketch of the new beamline layout in Fig. 1 ▸.

The parameters of the optics are displayed in Table 1 ▸. Here the width and length of the outer dimension, the active area on the surface of the mirror or grating, and the expected beam spot size on the optical element are tabulated. The slope errors are shown for both meridional and sagittal dimensions if different. The meridional slope errors have been measured in-house by BESSY-NOM (Siewert *et al.*, 2004 ▸, 2016 ▸). The sagittal slope errors were measured by the manufacturer, excluding the slope error for M5 which has been measured in-house. All optical elements have a reflective gold coating. Note that the horizontal aperture describes the aperture for the horizontally focused beam where the wide gap is along the vertical and vice versa for the vertical aperture.

The Apple-2 type undulator UE112 provides circular, elliptical or linear polarized light under various angles in the energy range from 17 eV to 690 eV. It consists of 32 periods of magnets at a period length of 112 mm. However, at lower energies polarization effects increase due to the reflection at the optical elements in the beamline. This can be compensated by a matching depolarization from the undulator as shown by Bahrdt *et al.* (2010 ▸) on a case to case basis, but this is rarely used. To suppress higher-order radiation from the undulator at lower energies, an Al or Mg foil can be introduced *in situ*. These act as low pass filters to suppress higher orders above 73 eV for Al and 50 eV for Mg. Additionally, the *c*_ff_ value of the mono can be adjusted to decrease higher-order transmission as described below. Note that the fixed-focus magnification factor *c*_ff_ = cosβ/cosα (with α and β being the incident and diffracted beam angles to the normal) also influences the flux and energy resolution of the beamline (Follath, 2001 ▸).

The toroidal mirror M1 collimates the beam from the undulator in the vertical plane for the monochromator and focuses horizontally on the horizontal aperture (Follath & Senf, 1997 ▸; Follath *et al.*, 1998 ▸; Follath, 2001 ▸). Due to the high heat load from the undulator radiation, the mirrors M1, M2 and the grating of the monochromator are water-cooled. The illumination of M1 can be adjusted by four water-cooled front-end apertures which are placed 3.2 m before M1.

The Petersen type monochromator (M2 and grating) disperses the beam vertically (Petersen, 1982 ▸; Domke *et al.*, 1992 ▸; Follath *et al.*, 1998 ▸). There are three laminar gratings available with line densities of 300 lines mm^−1^, 600 lines mm^−1^ and 1400 lines mm^−1^ which can be switched *in situ*. Owing to its variable *c*_ff_ one can choose between high flux (*c*_ff_ closer to 1), high energy resolution (*c*_ff_ away from 1), efficient higher-order suppression or zero order for alignment purposes (Follath & Senf, 1997 ▸).

The cylindrical mirror M3 focuses the collimated vertical light on the vertical aperture (exit slit). The intermediate focus positions at the horizontal and vertical slits are then further demagnified by the following refocusing optics M4 and M5. To reach such a strong demagnification, they are implemented as plane elliptical mirrors in a Kirkpatrick–Baez geometry (Kirkpatrick & Baez, 1948 ▸).

The focus point of the beamline lies 500 mm behind the centre of the last mirror. The outgoing beam is tilted upwards by 4°. The photoemission current of both refocusing mirrors can be measured. The mirror current of M5 is usually used to normalize against changes in photon flux. The sample chamber is also tilted by 4° and can be translated in all three dimensions^1^ . The sample holder is fixed in the chamber centre but can be rotated around the centre axis of the chamber. A cryogenic cooling stage is available.

As the beam arrives at an altitude angle of 4° on the sample which is tilted towards the spectrometer by 45°, there is an increase of the vertical beam width when observed from the spectrometer which, for practical reasons, is not tilted by 4°. The expected loss in energy resolution compared with a horizontal exit beam path is 3.5%.

The refocusing part of the beamline design was chosen as a test bed for an automated self-alignment and diagnostics framework. Therefore, all rotation axes of the refocusing mirrors have been motorized. We performed automated knife-edge scans in both the vertical and the horizontal directions to produce a labelled dataset of beam positions depending on predefined relative misalignments of the refocusing optics. The goal is that the absolute misalignments and therefore the best alignment can be deduced by a machine learning algorithm from these datasets. For this a neural network has been trained using simulated data from the ray-tracing software *RAY-UI* (Baumgärtel *et al.*, 2016 ▸, 2019 ▸).

## 3. Spectral resolution and flux

The energy resolution of the beamline was determined by measuring the photoionization current from the 2,1_4_ resonance of helium near 64 eV in a gas cell (Schulz *et al.*, 1996 ▸). The beam enters the cell through a polyamide window. Inside the cell a constant gas pressure of 1 × 10^−3^ mbar is maintained and a voltage of 300 V is applied between two electrodes to measure the photoionization current. The resolving power for the 300 lines mm^−1^ grating at a *c*_ff_ value of 7, an exit slit size of 10 µm and a vertical front-end aperture opening of 1 mm is better than 10000 or 6.36 meV at 64 eV. At lower *c*_ff_ values of 2.25 the energy resolution is 9.7 meV at 64 eV.

**Figure 2:**
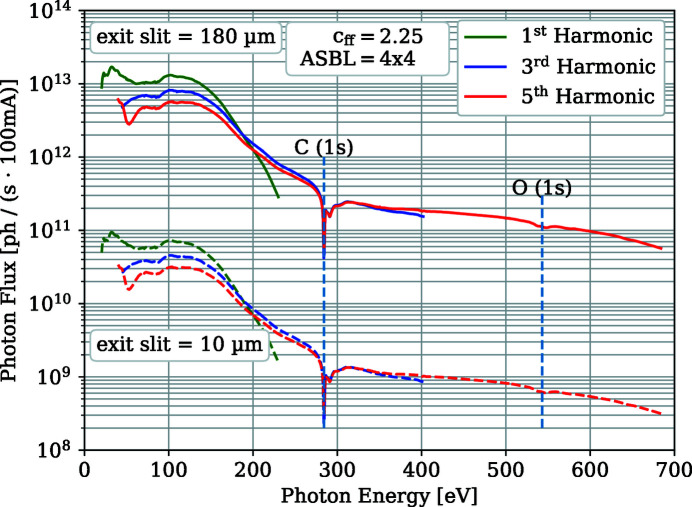
Photon flux of the beamline for horizontally polarized light and the first, third and fifth harmonics of the undulator with the 300 lines mm^−1^ grating at *c*_ff_ = 2.25 and open front-end apertures. The dashed lines indicate the flux measured at an exit slit of 10 µm. Note that, for energies below 50 eV, the photon flux has an increasing contribution of higher harmonics which can be suppressed with an Al or Mg foil.

**Figure 3:**
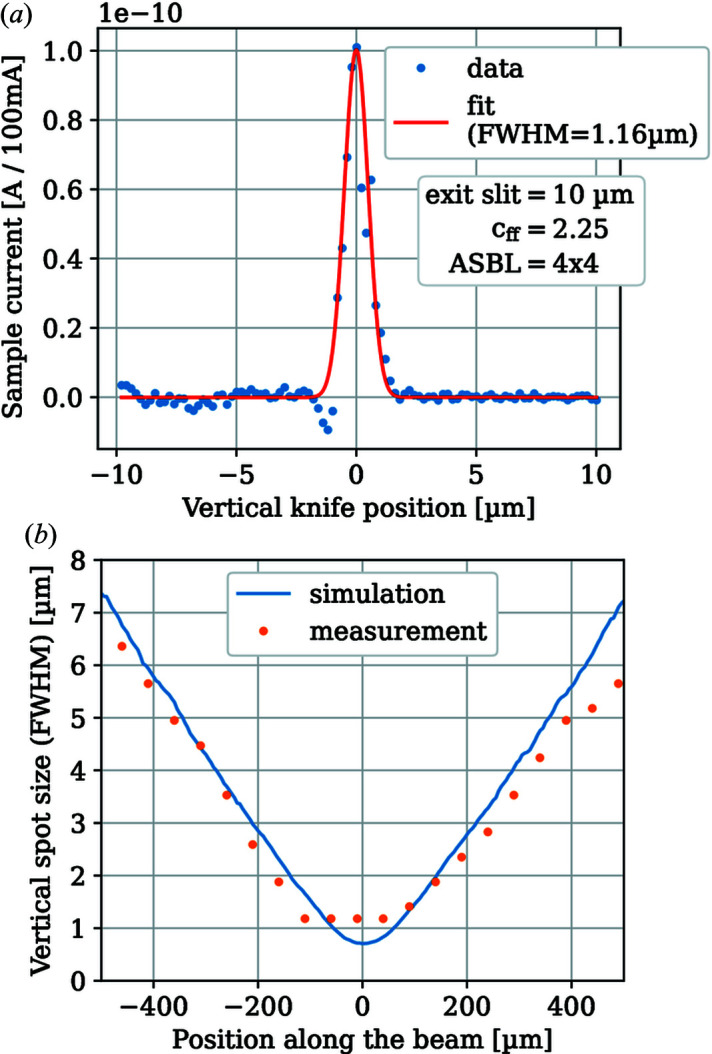
(*a*) Vertical beam profile at the sample position. Shown is the differentiated data from knife-edge scans. The positive (right-hand) side is the low signal (low noise) part of the scan. (*b*) Vertical beam size (FWHM) along the beam measured with knife-edge scans (dots) and simulated using *RAY-UI* (line).

The photon flux of the beamline is shown in Fig. 2 ▸. It was measured using a GaAsP diode just before the sample position using the 300 lines mm^−1^ grating of the monochromator at a *c*_ff_ value of 2.25 and opened front-end and exit slit. The beamline reaches a photon flux of 10^13^ photons s^−1^ (100 mA)^−1^ in the energy range from 17 eV to 150 eV and around 10^11^ photons s^−1^ (100 mA)^−1^ above the carbon edge of 284 eV up to 500 eV. This is similar to the previous beamline design which had the same number of optical elements and the same monochromator (Schiwietz *et al.*, 2015 ▸). We also included the calculated flux for an exit slit of 10 µm in Fig. 3 ▸.

## 4. Focus size and divergence

The size and position of the beam spot at the sample position were measured by knife-edge scans of the beam profile. The Au-coated knifes were mounted on a regular sample holder. The differentiated current profile along the vertical direction on the focus plane is shown in Fig. 3 ▸(*a*). Note that the knife-edge scan only gives an upper limit for the beam spot size due to irregularities of the edge itself. Here this problem is exaggerated due to the relatively high aspect ratio of the beam spot. A vertical focus size of 1.16 µm (FWHM) as well as a horizontal focus size (FWHM) of 20 µm have been measured. The divergence of the beam vertically around the focus position is shown in Fig. 3 ▸(*b*) and is in excellent agreement with the simulated beam profile using the design parameters of the mirrors and *RAY-UI*.

The vertical beam width is at or below 1 µm for about 100 µm along the beam. The horizontal beam width is far less divergent and can be assumed flat at this scale.

## 5. meV-RIXS spectrometer

Owing to the low count rates expected for RIXS measurements, the main design goals of the spectrometer were high transmission and long-term stability next to a high energy resolution. To achieve excellent long-term stability of the sample holder, the translation of the sample in all three directions is achieved by the translation of the whole sample chamber. A load lock for fast sample transfer or *in situ* storage of one sample is available.

**Figure 4:**
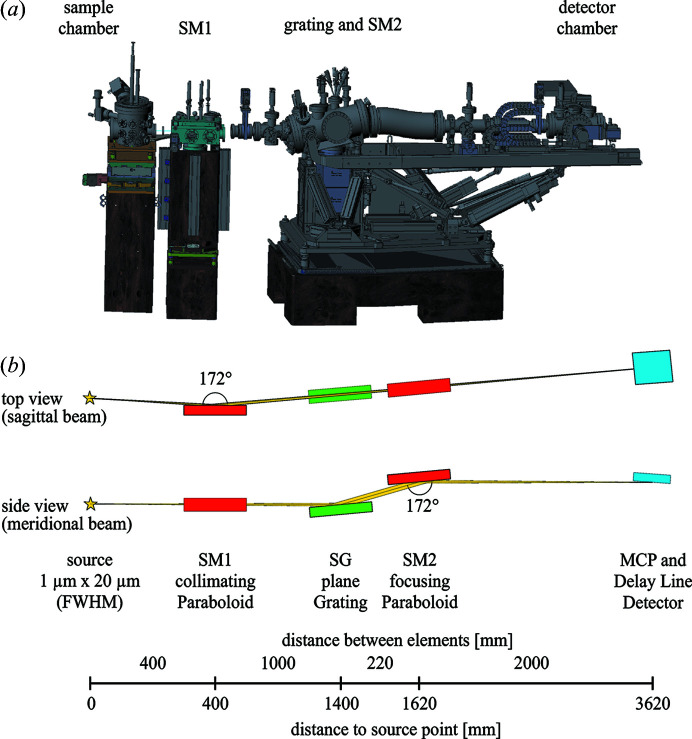
(*a*) Sketch of the sample chamber and meV-RIXS spectrometer. (*b*) Optical layout of the meV-RIXS spectrometer. Distances are design parameters and are given in millimetres.

A sketch and the optical layout of the spectrometer are shown in Fig. 4 ▸. The meV-RIXS spectrometer consists of two parabolic mirrors for collimation and focusing and a plane grating for dispersing the light. The spectrometer has horizontal and vertical apertures between SM1 and SG as well as between SM2 and the detector. The first set of apertures between SM1 and SG are used to block part of the beam originating from the edges of SM1 to reduce artefacts on the detector for high-resolution measurements. This is possible as the beam is collimated in this part of the spectrometer. However, the difference in flux between closed apertures for best energy resolution and fully open apertures is a factor of 10. Therefore, a compromise between minimizing artefacts and signal to noise considerations needs to be made on a case to case basis. The properties of the optical elements are listed in Table 2 ▸. All optics have been manufactured by Carl Zeiss Optronics GmbH and coated with Au.

The transmission of the instrument is mainly determined by the solid angle of the first optical element. Therefore, the first parabolic mirror is placed as close as possible to the sample. It has a focus length of 400 mm and captures a solid angle of 3 msr. There is no entrance slit between this mirror and the sample.

The energy resolution depends on the line density of the grating and the exit arm length after the grating. While the exit arm length is mainly limited by the available space, the line density of the grating is limited by the production process. By employing a plane grating instead of a spherical grating one can achieve higher line densities with lower slope errors of the optic. However, higher line densities also diffract less light. Therefore, we have two laminar gratings, a 1050 lines mm^−1^ with a groove depth of 10.5 nm and a 4200 lines mm^−1^ with a groove depth of 12 nm installed, which can be switched *in situ*. The 4200 lines mm^−1^ grating can be used for photon energies above 100 eV. Furthermore, a plane grating allows for adjusting the magnification factor of the spectrometer *c*_S_ and therefore the trade-off between energy resolution, transmission and higher-order suppression of the spectrometer. The definition of the *c*_S_ value is identical to the definition of the *c*_ff_ value for the monochromator, but since the fixed focus condition of the SX700 is not fulfilled here and to distinguish the two parameters it is henceforth called *c*_S_.

**Figure 5:**
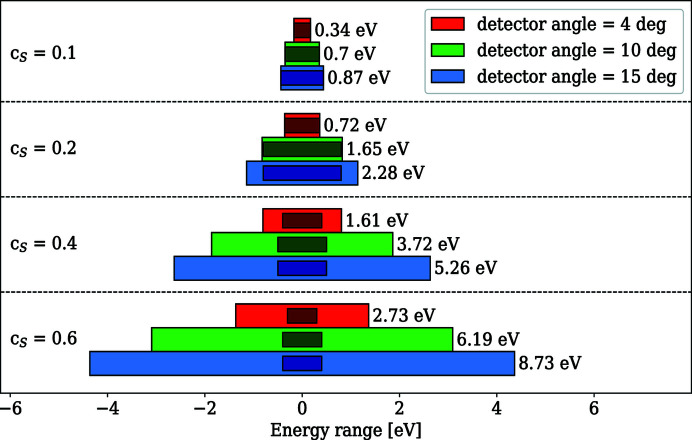
Simulated energy ranges of the spectrometer at 100 eV using the 1050 lines mm^−1^ grating for three angles at different *c*_S_ values. The smaller bar in the middle of each range indicates the range where the energy resolution d*E* is within a factor of 2 of its lowest value.

The second parabolic mirror with a focal length of 2 m focuses the diffracted light from the grating on the detector. The detector consists of a multi-channel plate (MCP; Photonis, 35671, Z-Stack) array and a custom made 2D delay-line detector from RoentDek Handels GmbH. The MCP has an open area ratio of 45%, an active area of 93 mm × 75 mm and a CsI coating to enhance sensitivity. As the second parabolic mirror produces a point focus we have a line along the vertical diffraction axis on the detector. This allows us to change the exposed area and therefore enhance the lifetime of the MCP by shifting the detector assembly horizontally. The detector chamber is attached to an arm where the rotation axis is positioned at the grating rotation axis. To follow the focal spot movement of the refocusing paraboloid for different energies, the detector is tilted to a grazing angle of 4°. However, due to the low vertical divergence of the beam at the focal point, steeper detector angles are possible to obtain a wider energy range on the detector without sacrificing the energy resolution significantly. The maximum detector angle of 15° is limited by the shadowing of radiation by the repeller grid bracket. Accessible energy ranges for *c*_S_ values smaller than 1 are shown in Fig. 5 ▸. The inner bars show the energy regions where the energy resolution is within a factor of 2 of the best value. These values were determined using *RAY-UI* at a spectrometer pass energy of 100 eV. Note that it is also possible to operate at *c*_S_ > 1 for even larger energy ranges as shown in Fig. 9.

The theoretical transmission and energy resolution dependence of the *c*_S_ value is shown in Fig. 7 of Könnecke *et al.* (2013 ▸). Note that the resolution *E*/d*E* of about 10 000 at 100 eV for the 1050 lines mm^−1^ grating mentioned there only relates to the grating resolution and does not take the slope errors of the parabolic mirrors nor the energy width of the beamline radiation into account. However, by taking the parabolic mirrors into account, the calculated energy resolution of the spectrometer alone is about 7700 for a *c*_S_ value of 0.05. With a beamline resolution of 10000 this results in a theoretical combined energy resolution of about 6100 at 100 eV for the 1050 lines mm^−1^ grating.

The stability of the beamline and spectrometer has been checked by continuously measuring the position of the elastic line of NiO at 60 eV. The peak position stayed within ±1.25 meV for 5 h. The energy resolution was 30 meV FWHM.

**Figure 6:**
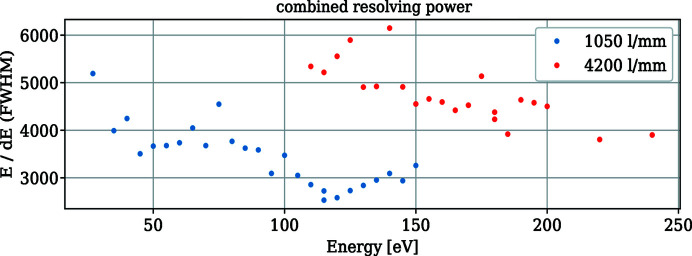
Measured combined resolving power of the beamline and spectrometer at *c*_S_ = 0.05 and a detector angle of 4°. The spectrometer pass energy has been adjusted for each data point and the energy calibrations were performed using the elastic peak from NiO.

The measured combined resolving powers of the beamline and spectrometer for both gratings are shown in Fig. 6 ▸. Here we used the elastic peak of an NiO sample and the beamline monochromator energy to perform an energy calibration for each pass energy of the spectrometer at a constant *c*_S_ value of 0.05. An example of this procedure and the elastic peak shape are given in the supporting information. The *c*_ff_ value of the monochromator was 2.25. The exit slit and front-end aperture gap of the beamline were 5 µm and 1 mm at 100 eV but had to be increased up to 20 µm and 2 mm for energies lower than 90 eV and higher than 140 eV.

The resolution *E*/d*E* of about 3500 at 100 eV for the 1050 lines mm^−1^ is lower than the calculated resolution of 6100. While we cannot completely rule out misalignments of the spectrometer, it is likely that the slope errors of the parabolic mirrors are higher than anticipated. Due to the high curvatures of these mirrors, we were only be able to measure the meridional slope errors along the centre of the mirrors. The off-axis meridional slope errors and the sagittal slope errors could not be confirmed independently. The fact that we had to close the apertures between the first parabolic mirror SM1 and the grating SG to reduce artefacts of the beam spot on the detector and increase the energy resolution supports this theory. The shape of the artefacts mimic the beam spot shape originating from the off-axis areas along SM1 as calculated using *RAY-UI*. They disappear when the apertures block the radiation from the off-axis areas. However, as the radiation between SM1 and SM2 is collimated, both mirrors are only partly irradiated when we close the apertures, which makes it difficult to determine which mirror is the cause of the artefacts.

**Figure 7:**
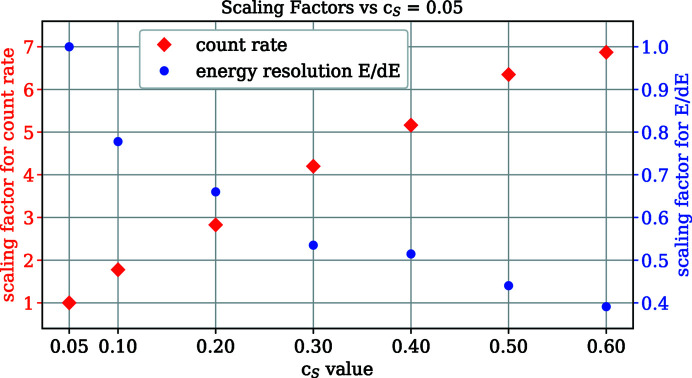
Scaling of the transmission and energy resolution as a function of *c*_S_ value. The FWHM of the elastic line from Au at 100 eV and the sum of the count rates over this peak were used to determine these values. The detector angle was 15°.

Fig. 7 ▸ shows the measured energy resolution and flux as a function of the *c*_S_ value.

The measurement was taken with the elastic line from Au at 100 eV using linear vertical polarization and an exit slit of 10 µm to minimize the impact of the beamline resolution.

**Figure 8:**
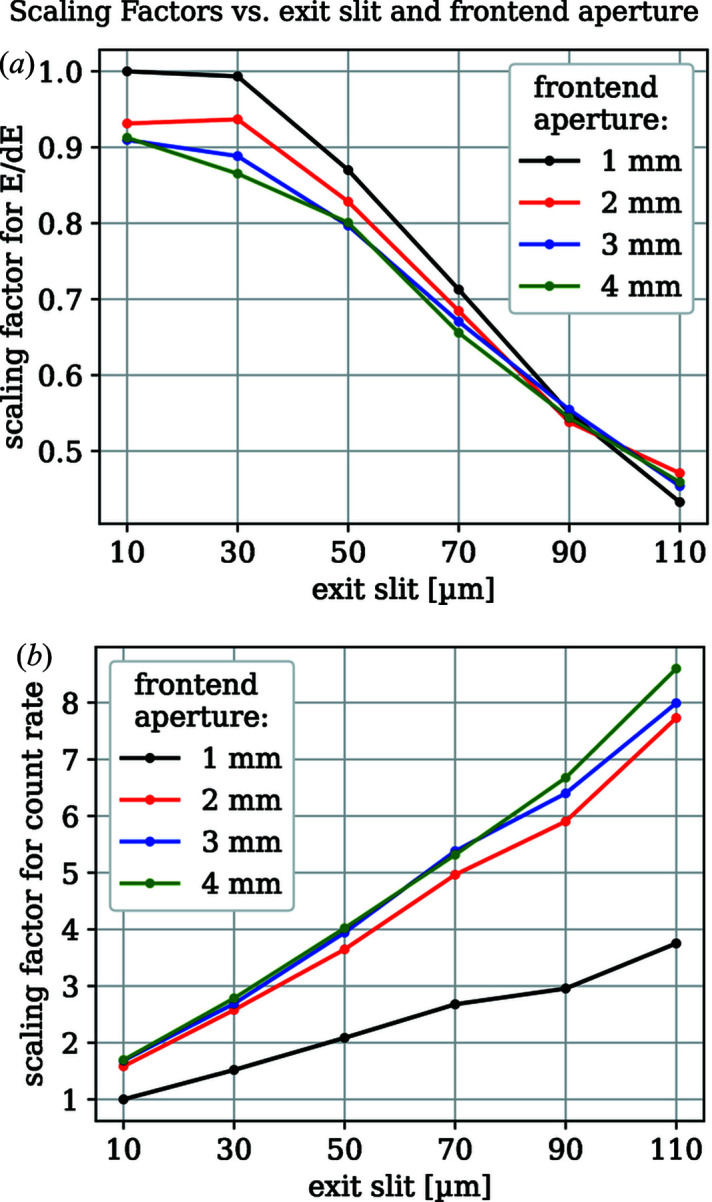
Scaling of the transmission and energy resolution as a function of the front-end aperture and exit slit sizes. The front-end aperture size given here is the vertical and horizontal width of the gap. The FWHM of the elastic line from hBN at 192 eV and the sum of the count rates over this peak were used to determine these values. The *c*_S_ value was 0.3 and the detector angle was 4°.

The measured energy resolution of the spectrometer is not only dependent on spectrometer parameters but also on the energy width of the beamline radiation and the spot size on the sample. The beamline resolution is influenced by both the exit slit size and the front-end aperture size at a given energy and *c*_ff_ value. The spot size is mainly influenced by the exit slit. Fig. 8 ▸ shows the scaling factors of the combined energy resolution and flux at the spectrometer for these beamline parameters. Here the *c*_ff_ value is 2.25. We used the elastic line at 192 eV at the B *K*-edge of hBN at a *c*_S_ value of 0.3 and a detector angle of 4°. The front-end size is the width and height of the gap that is formed by the front-end apertures. As the aperture blades from the top, bottom, right and left are independent, one can also only close the top and bottom apertures, since the energy resolution depends mainly on them. This will alter the scaling factor of the count rate.

## 6. Rowland-type spectrometer

In Section 5 we show that the energy range of the meV-RIXS spectrometer can be adjusted by changing the *c*_S_ value and the detector tilt. However, for novel compounds especially, it is crucial to be able to perform even wider energy range measurements on a short time scale to identify areas of interest before investing several hours on high-resolution spectra acquisitions. While it is possible to stitch several smaller spectra from meV-RIXS together, this multiplies the measurement time by the number of spectra needed.

**Figure 9:**
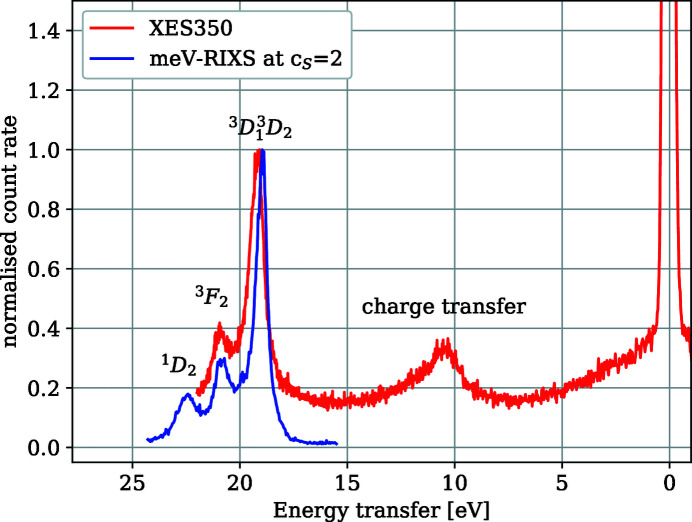
Spectra of SrLaAlO_4_ using the low-resolution Rowland-type spectrometer XES350 and meV-RIXS at a *c*_S_ value of 2 and a detector angle of 15°. The incidence energy was 101.8 eV. The energy ranges of the meV-RIXS and XES350 are 8.75 eV and 23 eV, respectively. The spectra have been normalized to the ^3^ *D*_1_^3^ *D*_2_ peak.

Therefore, a compact spectrometer with a wider energy window is attached to the sample chamber on the opposite position of the meV-RIXS spectrometer. By rotating the sample, one can switch between both spectrometers. It utilizes a Rowland-type geometry similar to a VG Scienta XES350^2^ spectrometer, which is based on the Nordgren design (Nordgren *et al.*, 1989 ▸). This spectrometer includes three different gratings (listed in Table 3 ▸) and the delay line detector DLD 4040 from Surface Concept GmbH. It allows us to carry out fast overview spectra and define the region of interest for high-resolution measurements using the meV-RIXS spectrometer. An example of the different energy ranges and spectrometer performances of the two spectrometers is shown in Fig. 9 ▸ for SrLaAlO_4_. Here the energy range of the meV-RIXS spectrometer has been increased close to its maximum using a *c*_S_ value of 2 and a detector angle of 15°.

We chose an excitation energy at the ^3^ *D*_1_ resonance of La at 101.8 eV.

SrLaAlO_4_ was chosen primarily for two reasons: (1) as for other reported La-based compounds, for an excitation at the La 3*D*_1_ pre-resonance at 101.8 eV, one observes a multipeak fine structure (Suljoti *et al.*, 2009 ▸), which can be resolved with meV-RIXS; (2) this multipeak feature occurs at around 20 eV energy loss, close to the energy range limit of the overview spectrometer at this excitation energy.

The energy calibration for both spectra was performed independently using the elastic line of the monochromator energy. The meV-RIXS spectrum was taken at a monochromator *c*_ff_ value of 2.25, an exit slit of 100 µm and a front-end aperture opening of 4 mm × 4 mm. The XES350 spectrum was obtained at the same *c*_ff_ value but reduced flux at an exit slit of 50 µm and a front-end aperture of 2.4 mm × 2.4 mm. The acquisition time for both spectra was 20 min. While the meV-RIXS spectrum has an 8.75 eV energy range and an 85 meV to 350 meV energy resolution, the XES350 spectrum is 23 eV wide with a 413 meV energy resolution. For this measurement we used the 400 lines mm^−1^ grating of the XES350. Note that by using a similar spectrometer mounted on a different setup, an energy range of 40 eV for a 143.5 eV excitation energy can be reached (Decker *et al.*, 2021 ▸).

Even in wide energy range configuration, only the multipeak feature is visible in a single spectrum with meV-RIXS. On the contrary, three spectral features, namely the multipeak feature, a second feature at 10 eV loss attributed to a charge transfer excitation (Moewes *et al.*, 2000 ▸) and the zero-loss elastic peak, are visible in a single spectrum with the overview spectrometer XES350.

## 7. Control software

We operate both the beamline and the meV-RIXS spectrometer using Python scripts in a Jupyter notebook environment (https://jupyter.org/). The underlying custom framework JupyterBLC uses EPICS (https://epics.anl.gov/, https://github.com/pyepics/pyepics) and direct serial connections to communicate with various actors and sensors and allows real-time plotting and analysis of generic 1D and 2D measurements. Furthermore, the detector images and spectra as well as the optical camera images are stored and processed in these notebooks. Therefore, they also act as a digital laboratory book utilizing markdown cells. It is possible to run the instrument remotely or perform typical tasks automatically using this framework. The full digitalization of experiment control, data storage and data processing opens the route to future beamline optimization techniques using machine learning protocols, which are currently under development at BESSY II.

## 8. Summary

The UE112-PGM1 beamline at BESSY II has been rebuilt to provide a micrometre focus in the vertical dimension for the meV-RIXS spectrometer in the energy range from 17 eV to 690 eV. By employing plane elliptical refocusing KB optics a focus size of 1.16 µm × 20 µm (vertical × horizontal, FWHM) was reached. The resolution of the beamline was found to be better than 10000 at 64 eV and better than 6500 at an increased photon flux of 1 × 10^13^ photons s^−1^ (100 mA)^−1^. The measured combined energy resolution of the beamline and spectrometer is 3000–6000 in the range from 60 eV to 240 eV. This will allow RIXS measurements of transition metal *M*-edges, rare earth *N*-edges and the *K*-edges of light elements up to carbon on surfaces. A separate Rowland-type spectrometer was attached to the sample chamber to facilitate fast low-resolution and large energy range scans to gather a quick overview of the peak positions in novel materials.

## Supplementary Material

Figure S1. DOI: 10.1107/S1600577522003551/ok5067sup1.pdf

## Figures and Tables

**Table 1:** Properties of the optical elements of the beamline The width and length are given in ‘outer size/active area/expected spot size’ and the slope errors are given in meridional and sagittal beam directions if they differ.

Element	Type	Width (mm)	Length (mm)	Incidence angle (°)	Slope error (arcsec)
UE112	Apple-2 undulator				
M1	Toroid mirror	40/20/2	200/180/80	3	0.1/1
M2	Mono premirror	30	300	Variable	0.1
G	Mono plane grating	20	100	Variable	0.1
M3	Cylindrical mirror	40/20/4	200/180/40	1.5	0.06/0.8
A4	Horizontal aperture				
A5	Vertical aperture				
M4	Plane ellipse	40/20/10	200/180/200	1.5	0.09/0.18
M5	Plane ellipse	40/20/4	300/280/200	2	0.034/0.142

**Table 2:** Properties of the optical elements of the meV-RIXS spectrometer The width and length are given in ‘outer size/active area’ and the slope errors are given in meridional and sagittal beam directions.

Element	Type	Width (mm)	Length (mm)	Azimuthal angle (°)	Slope error (arcsec)
SM1	Parabolic mirror	40/35	200/180	90	3.51/1.89
SG	Plane grating	40/20	200/180	0	0.13/0.3
SM2	Parabolic mirror	60/20	300/270	180	0.5/2.3

**Table 3:** Blazed spherical grating properties of the Rowland-type spectrometer All gratings have a 30 nm reflective Au coating. All values are design parameters from the manufacturer.

Line density (lines mm^−1^)	400	600	2400
Radius	3000	5000	5000
Energy range (eV)	>20	55–600	250–1350
Optimized energy (eV)	100	275	350
Blaze angle (°)	1.0	0.95	2.0

## Footnotes

1
*XY* stage 5102.50-019 and *Z* stage 5103.c40-014 are made by HUBER Diffraktionstechnik GmbH & Co. KG, Rimsting, Germany.

2 Formerly known as GRACE IV, Scienta merged with Omicron in 2015.
