# Supplementary material for: The meV XUV-RIXS facility at UE112-PGM1 of BESSY II
Source: J Synchrotron Radiat. 2022 Apr 26;29(Pt 3):908–15. doi: 10.1107/S1600577522003551 (PMC9070711; doi:10.1107/S1600577522003551)
Supplement: Supplementary file 1 [file s-29-00908-sup1.pdf]

JOURNAL OF SYNCHROTRON RADIATION

Volume 29 (2022)

Supporting information for article:

The millielectronvolt XUV-RIXS facility at UE112-PGM1 of BESSY II

Karl Bauer, Jan-Simon Schmidt, Frank Eggenstein, Régis Decker, Kari Ruotsalainen, Annette Pietzsch, Thomas Blume, Chun-Yu Liu, Christian Weniger, Frank Siewert, Jana Buchheim, Grzegorz Gwalt, Friedmar Senf, Peter Bischoff, Lisa Schwarz, Klaus Effland, Matthias Mast, Thomas Zeschke, Ivo Rudolph, Andreas Meißner and Alexander Föhlisch

Figure 1

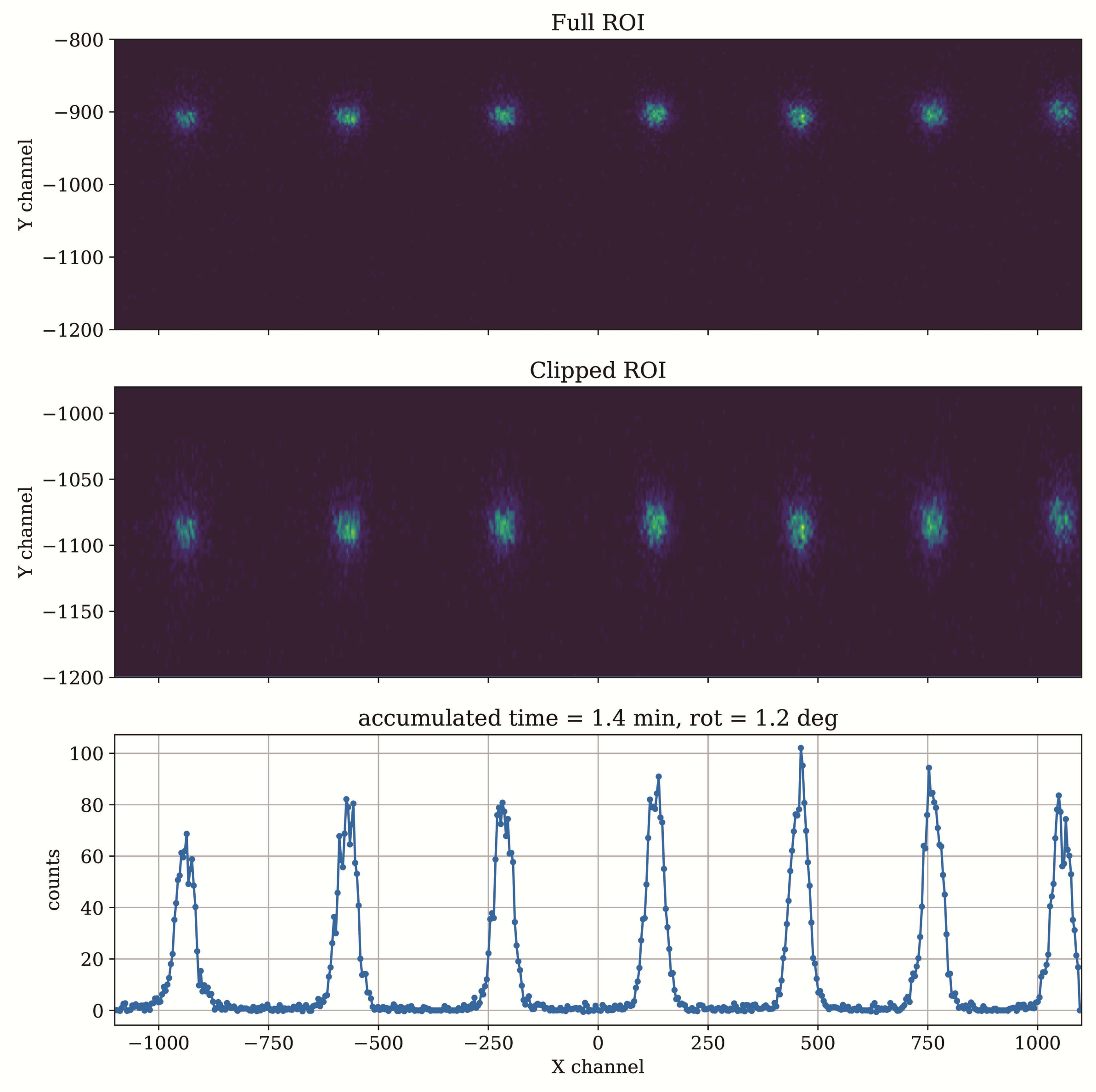
Full ROI
−800
−900
−1000
−1100
−1200
Y channel
Clipped ROI
−1000
−1050
−1100
−1150
−1200
Y channel
accumulated time = 1.4 min, rot = 1.2 deg
100
80
60
40
20
0
counts
−1000
−750
−500
−250
0
250
500
750
1000
X channel
